# Regional Anaesthesia in the Patient with Pre-Existing Neurological Dysfunction

**Published:** 2009-04

**Authors:** Pramila Bajaj

**Affiliations:** Editor, IJA

Patients with pre-existing neurological disease present a unique challenge to the anaesthesiologist. The cause of postoperative neurological deficits is difficult to evaluate, because neural injury may occur as a result of surgical trauma, tourniquet pressure, prolonged labour, or improper patient positioning or anaesthetic technique. Progressive neurological diseases such as multiple sclerosis may coincidentally worsen perioperatively, independently of the anaesthetic method. The most conservative legal approach is to avoid regional anaesthesia in these patients. However, high-risk patients, including those with significant cardiopulmonary disease, may benefit medically from regional anaesthesia and analgesia. The decision to proceed with regional anaesthesia in these patients should be made on a case-by-case basis. Meticulous regional anaesthetic technique should be observed to minimise further neurological injury.

## Risk factors for regional anaesthesia-related nerve injury

Neurological injury directly related to regional anaesthesia may be caused by trauma, neurotoxicity and ischaemia. Direct needle- or catheter-induced trauma rarely results in permanent neurological injury. The overall incidence of persistent paraesthesias has been estimated at 0.08% after spinal anaesthesia and at 2% after brachial plexus block^1^,^2^. It has been suggested that paraesthesia techniques may be associated with a higher incidence of neurological injury after brachial plexus block, but there are no conclusive data supporting that claim^2^.

The needle-bevel configuration may influence the frequency and severity of peripheral nerve damage during regional anaesthesia. In an in vitro study, Selander et al^3^ demonstrated an increased frequency of perineural injury when a long-bevelled need was used instead of a short-bevelled needle. Rice and McMahon^4^ assessed frequency and severity of neural trauma after nerve impalement by histological and clinical methods and reported that injury produced by short-bevelled needles was more severe and more frequent, and that recovery from it was slower than in the case of injury produced by long-bevelled needles. Although no human studies have been performed to determine which of these in vitro studies accurately predicts clinical outcome, these studies illustrate the importance of minimising direct needle trauma during regional techniques, especially in patients at increased risk of neurological complications.

Neurological deficits after regional anaesthesia may be a direct result of local anaesthetic toxicity. Clinical and laboratory findings indicate that anaesthetic solutions are potentially neurotoxic^5^–^9^. It is generally agreed that local anaesthetics administered in clinically appropriate doses and concentrations do not cause nerve damage^10^. However, prolonged exposure to high concentrations of local anaesthetic solutions may result in permanent neurological deficits. Patients with underlying nerve dysfunction may have a decreased requirement for local anaesthetic and a decreased threshold for neurotoxicity^7^. Indeed, Yee et al^11^ have demonstrated that the dose requirement for local anaesthetics is decreased and potency increased in aged animals. This may have implications for the use of local anaesthetics in an ageing patient population.

Neural ischaemia may occur as a result of systemic or local vascular insufficiency. Systemic hypotension with or without a spinal anaesthetic may produce spinal cord ischaemia in the watershed areas between radicular vessels, resulting in flaccid paralysis of the lower extremities (anterior spinal artery syndrome). The use of local anaesthetic solutions containing epinephrine or phenylephrine may theoretically result in local ischaemia, especially in patients with microvascular disease, but clinical data are lacking^8^,^12^. Furthermore, large clinical studies have failed to identify the use of vasopressors as a risk factor for neurological injury. Most cases of presumed vasopressor-induced neurological deficits after spinal anaesthesia have been single case reports, often with several other risk factors involved^13^.

## Epidural and spinal anaesthesia after major spinal surgery

Previous spinal surgery has been considered to represent a relative contraindication to the use of regional anaesthesia. Many of these patients experience chronic back pain and are reluctant to undergo epidural or spinal anaesthesia, fearing exacerbation of their pre-existing back complaints. Several postoperative anatomical changes make needle or catheter placement more difficult and complicated after major spinal surgery. In a study 105 of 48 patients with chronic low back pain after spinal fusion, 8 showed significant spinal stenosis on computed tomographic scans and requited surgical decompression^14^. The ligamentum flava may be injured during surgery, resulting in adhesions within or obliteration of the epidural space. The spread of epidural local anaesthetic maybe affected by adhesions, producing an incomplete or ‘patchy’ block. Obliteration of the epidural space may increase the incidence of dural puncture and make subsequent placement of an epidural blood patch difficult. Needle placement in an area of the spine that has undergone bone grafting and posterior fusion is not possible with midline or lateral approaches; needle insertion can be accomplished at unfused segments only.

The guidelines for epidural anaesthesia after spinal surgery are unclear. Daley et al^15^ reviewed the charts of 18 patients with previous Harrington rod instrumentation who underwent 21 attempts at epidural anaesthesia for obstetric analgesia. Continuous lumbar epidural anaesthesia was successfully established in 20 of 21 attempts, but only 10 procedures were performed easily at the first attempt. The remaining 11 patients required larger amounts of local anaesthetics or complained of a patchy block or both. There was no correlation between the level of surgery and the ease of insertion or the quality of epidural anaesthesia. There were no side-effects except for low back pain in two patients in whom multiple attempts at catheter placement had been necessary.

Crosby and Halpern^16^ studied nine parturients with previous Harrington rod instrumentation who underwent epidural anaesthesia for analgesia during labour and delivery. Five of the nine catheters were successfully placed at the first attempt. Four of the nine procedures were complicated and involved multiple attempts before successful insertion, traumatic catheter placement requiring a second insertion, inadequate epidural analgesia with subsequent dural puncture on a repeated attempt or inability to locate the epidural space despite attempts at two levels. Seven of the nine patients obtained satisfactory analgesia. There were no adverse sequelae related to the epidural insertion.

Hubbert^17^ described attempted epidural anaesthesia in 17 patients with Harrington rod instrumentation. Four of five patients with fusions terminating above the interspace between L-3 and L-4 had successful epidural placement. However, in 12 patients with fusions extending to the interspace between L-5 and S-1, 6 attempts were unsuccessful, 5 patients required multiple attempts, and 1 patient had a dural puncture after multiple attempts at epidural placement before it was successfully achieved. A false loss of resistance was reported to have occurred frequently.

Thus, historically it was concluded that epidural anaesthesia may be successfully performed in patients who have had previous spinal surgery, but successful catheter placement may be possible on the first attempt in only 50% of patients, even by an experienced anaesthesiologist. Although adequate epidural anaesthesia is eventually produced in 40-95% of patients, there appears to be a higher incidence of traumatic needle placement, unintentional dural puncture and unsuccessful epidural needle or catheter placement, especially if spinal fusion extends to between L-5 and S-1.

A more recent investigation examined the overall success and neurological complication rates among 937 patients with spinal stenosis or lumbar disc disease undergoing neuraxial block between 1988 and 2000^18^. Of these, 210 (22%) patients had a co-existing peripheral neuropathy in addition to their spinal cord pathology. Gender distribution was 619 (66%) male and 318 (34%) female. The mean age of these patients was 67±14 years. Neurological diagnoses had been known for a mean of 5±6 years; 335(51%) patients had active symptoms at the time of the block. In addition, 207 (22%) patients had a history of prior spinal surgery before undergoing neuraxial block, although the majority were simple laminectomies or discectomies.

Success rates did not differ between patients who had previous surgery and those who had undergone a spinal procedure. Ten (1.1%; 95% CI 0.5-2.0%) patients experienced new or progressive neurological deficits compared with preoperative findings. Although the majority of the deficits were related to surgical trauma or tourniquet ischaemia, the neuraxial block was the primary aetiology in 4 patients.

The preliminary nature of these data warrants care in their interpretation. However, overall, patients with spinal stenosis or lumbar disc disease may undergo successful neuraxial block without a significant increase in neurological complications. Importantly, this includes patients who have undergone prior (minor) spinal surgery.

## Anaesthetic management of neurological disease

Progressive neurological disease is considered by some to be a relative contraindication to regional anaesthesia, because of the difficulty in determining the cause of new neurological deficits that appear perioperatively. There are no controlled clinical studies identifying regional anaesthesia as a significant factor in increased risk of neurological injury, only anecdotal reports are available. The medicolegal issue, however, remains, and if regional anaesthesia is indicated for other pre-existing medical conditions or by patient request, the patient should be informed of the risk of neurological complications, including coincidental progression of preoperative deficits, associated with anaesthesia and surgery. This discussion, along with preoperative neurological status, should be fully documented in the patient's record.

Patients with preoperative neurological deficits may undergo further nerve damage more readily from needle or catheter placement, local anaesthetic systemic toxicity, and vasopressor-induced neural ischaemia. Although the use of paraesthesia techniques is not contraindicated, care should be taken to minimize needle trauma and intraneuronal injection. Dilute local anaesthetic solutions should be used whenever feasible to decrease the risk of local anaesthetic systemic toxicity.

The use of epinephrine-containing solutions is controversial. The potential risk of vasopressor-induced nerve ischaemia must be weighed against the advantages of predicting local anaesthetic intravascular injections, improved quality of block, and decreased blood levels of local anaesthetics. Because epinephrine also prolongs and block and therefore neural exposure to local anaesthetics, the appropriate concentration and dose of local anaesthetic solutions must be considered. Patients with microvascular disease in combination with an underlying peripheral neuropathy, such as those with diabetes, may be most sensitive to the vasoconstrictive effects of epinephrine.

Efforts should also be made to decrease neural injury in the operating room through careful patient positioning. Postoperatively, these patients must be followed closely to detect potentially treatable sources of neurological injury, including constrictive dressings, improperly applied casts and increased pressure on neurologically vulnerable sites. New neurological deficits should be evaluated promptly by a neurologist for formal documentation of the patient's evolving neurological status and the arrangement of further testing and long-term follow-up.
